# A highly sensitive modified nested PCR to enhance case detection in leishmaniasis

**DOI:** 10.1186/s12879-019-4180-3

**Published:** 2019-07-15

**Authors:** Bhagya Deepachandi, Sudath Weerasinghe, Preethi Soysa, Nadira Karunaweera, Yamuna Siriwardana

**Affiliations:** 1 Deparment of Parasitology, Faculty of Medicine, University of Colombo, Colombo, Sri Lanka; 2 Department of Biochemistry and Molecular Biology, Faculty of Medicine, University of Colombo, Colombo, Sri Lanka

**Keywords:** Leishmaniasis, Diagnosis, PCR, *L. donovani*

## Abstract

**Background:**

Human leishmaniasis is one of the major parasitic diseases with worldwide distribution. Sri Lanka is a recently established focus of leishmaniasis caused by a variant *Leishmania donovani*. Early case detection and management is a main approach identified for *L. donovani* control in the regional leishmaniasis elimination drive. Usefulness of light microscopy and in-vitro culture are limited in chronic, atypical or treated lesions though timely and accurate detection of all light microscopy/in-vitro culture negative cases of all forms of leishmaniasis is necessary for treatment. Timely treatment is important to minimize risk for death in visceral disease and undesired sequelae of long standing infection and illness on both patients and community. We described a 100% sensitive, *Leishmania* spp. specific modified version of a nested PCR (Mo-STNPCR) that also minimizes carry over and cross contaminations while facilitate investigation of light microscopy and in-vitro culture negative clinically suggestive cases of leishmaniasis.

**Methods:**

*Leishmania* DNA was amplified using previously published P221: 5′-GGTTCCTTTCCTGATTTACG-3′ and P332: 5′-GGCCGGTAAAGGCCGAATAG-3’outer primers followed by a nested reaction using P223: 5′-TCCCATCGCAACCTCGGTT-3′ and P333: 5′-AAGCGGGCGCGGTGCTG-3′ inner primers that by passes the requirement of tube handling between the two steps of the conventional nested PCR. *Leishmania* DNA was detected in a range of infected tissue material. Infected material from patients with cutaneous leishmaniasis (*n* = 30), visceral leishmaniasis (*n* = 10) and from a control group including patients with non-leishmanial skin diseases (*n* = 10), other systemic diseases (*n* = 10) and healthy individuals (*n* = 10) were examined with Mo-STNPCR. Results were further compared with those of light microscopy and in-vitro culture.

**Results:**

Mo-STNPCR method was 100% sensitive and 100% specific for diagnosis of leishmaniasis. Light microscopy and in-vitro culture were positive in 75.0% (*n* = 30/40) and 72.5% (*n* = 29/40) samples respectively where combined results of them gave 87.5% (*n* = 35/40) sensitivity. Mo-STNPCR did not cross react with control samples. Furthermore, Mo-STNPCR reduces the risk of cross-contaminations and carry over contaminations since the full reaction is carried out without opening the tubes. Per patient cost was calculated as 22 USD while the same was 3 and 6 USD for light microscopy and in-vitro culture respectively.

**Conclusion:**

Mo-STNPCR method is a useful tool in detecting leishmaniasis in minority of cases that go undetected by first line investigations.

## Background

All clinical forms of leishmaniasis are associated with high morbidity and also with mortality in the case of VL and MCL [1]. Human leishmaniasis continue to remain a major health issue in many countries despite many efforts in disease control [1]. There is a regional leishmaniasis elimination drive for *L. donovani* infections in the Indian subcontinent (ISC) that aims at achieving its targets by year 2020 [2].

Sri Lanka is a recent focus of human leishmaniasis in ISC with *L. donovani* being the causative agent [3–5]. So far, in a clear majority of reported local cases the disease is apparent in the form of CL [3, 6–8]. Early disease confirmation and management is considered very important in disease control of *L. donovani*. Recent emergence of VL and MCL [9–11], poor treatment response [12], micro changes within CL profile [13], widening case distribution [8], atypical manifestations [12, 13], regionally varied risk factors [14–16] and questionnable potential for visceralization [17] further necessitates urgent action in this locality. Detection of all infections and all clinically apparent cases are important. VL essentially requires pre-treatment laboratory confirmation of all cases.

Success rate of first line investigation, light microscopy (LM) depend on parasite load and technical handling though it is the cheapest, least complex, quick and field friendly. Low parasite counts in chronic and partially treated infections usually lead to false-negative results when traditional diagnostic assays are used. Therefore, more sensitive tools are required to detect those clinically suspected cases that turn negative in LM. In-vitro culturing (IVC) of parasites is complex, expensive and time consuming while reporting can take time depending on parasite growth in culture media. All *Leishmania* inoculations do not end up in successful parasite growth in artificial media. Molecular detection of *Leishmania* using PCR is the most sensitive method to date. However, molecular techniques are highly expensive, require sophisticated laboratory facilities and require technical expertise limit its usage for performance in resource poor endemic field areas [18]. However, availability of a highly sensitive PCR assay at a central laboratory would be extremely useful to establish an immediate diagnosis in microscopy negative minority that demands a quick diagnosis. Most researchers have shown PCR as a sensitive and specific tool for diagnosis of leishmaniasis and shown excellent correlations between PCR results and other diagnostic methods such as parasitological and serological tests. Conventional single-step PCR (CPCR), quantitative real-time PCR (qPCR), nested PCR (NPCR) and PCR-restriction fragment length polymorphism (PCR-RFLP) are widely used to detect different genetic sequences of *Leishmania* such as kinetoplastid DNA (kDNA), internal transcribed spacer (ITS) region, and small subunit ribosomal RNA (ssurRNA) [19–23].

PCR protocols and loop-mediated isothermal amplification (LAMP) have been tested locally [18, 24]. The LAMP is a relatively less complex method that is cost-effective and require low performance time [18]. But the low sensitivity rate of LAMP limits its use as a diagnostic tool. Furthermore, in the local settings *Leishmania* genus-specific kDNA and ITS1 PCR assays have demonstrated 92% sensitivity, while the sensitivity level of *L. donovani* species-specific kDNA assay is only 71% [24].

NPCR is a modification of CPCR and employs two sets of primers and two successive PCR reactions. Although the conventional nested PCR is one of the most sensitive PCR techniques, the possible cross-contaminations and carry over contaminations are the major difficulties associated [25]. They may occur during opening of reaction tubes during preparation for the second step amplification by transferring amplicons already produced during the first amplification step, subsequently result in false positives and therefore reduce the accuracy of the test. STNPCR described here involves both first and second PCR reactions of nested PCR, but both placed in one tube by immobilizing inner primers on inner side of cap of PCR tube and dissolving before the second round of PCR [25]. Therefore it reduces the possibility of cross-contaminations during procedure. The STNPCR methods have been proven useful in detection of a range of conditions [25–29]. STNPCR method has been proven useful in detection of low amounts of parasite DNA as well [25].

Present study evaluated the performance of a further modified nested PCR (Mo-STNPCR) method to achieve better sensitivity, specificity and to avoid possible cross-contamination and carry over contaminations associated with conventional NPCRs.

## Methods

### Primer selection

**Fig. 1:**
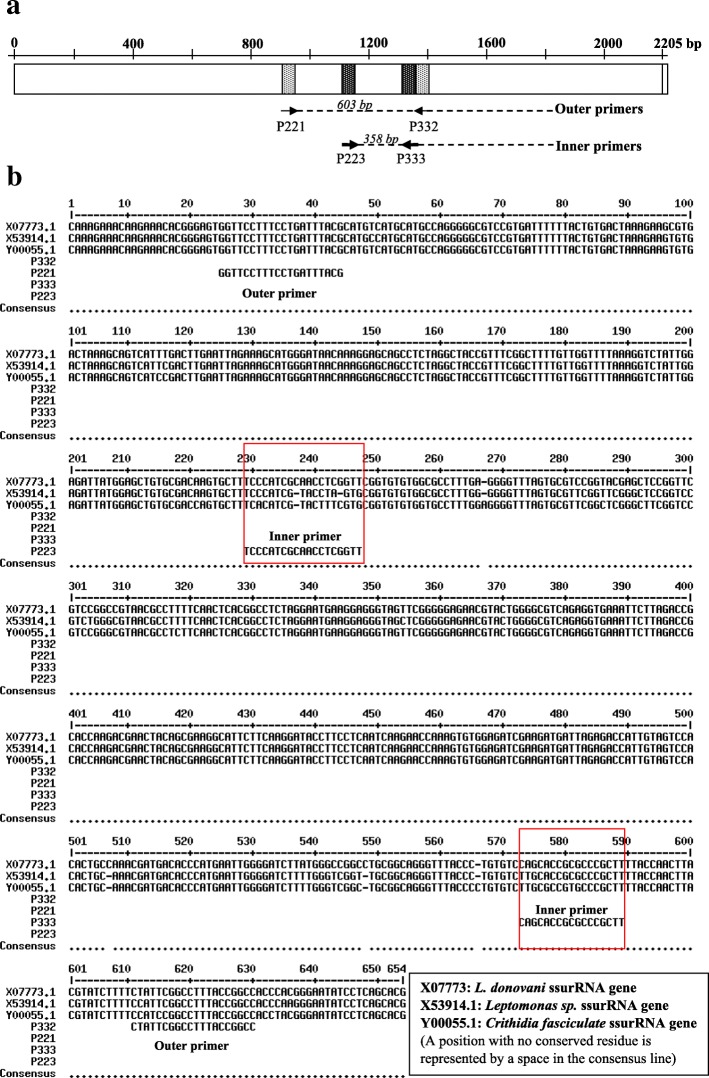
**a**: Annealing sites of P221-P332 and P223-P333 in *L. donovani* gene for ssurRNA (GenBank: X07773.1) [31]. **b**: Sequence difference of selected kinetoplastid organisms showing specificity of inner and outer primers [31]

Two pairs of primers, outer (P221: 5′-GGTTCCTTTCCTGATTTACG-3′ and P332: 5′-GGCCGGTAAAGGCCGAATAG-3′) and inner (P223: 5′-TCCCATCGCAACCTCGGTT-3′ and P333: 5′-AAGCGGGCGCGGTGCTG-3′) primers were employed (Fig. 1a) [18]. P221 and P332 primers were designed to amplify kDNA of genus *Leishmania*. Although P221 and P332 primers may amplify the other kinetoplastida such as *Leptomonas* and *Crithidia*, inner primers (P223 and P333) were designed as specific for *Leishmania* genus [30]. As published by Cruz et al*,* the sequence analysis of inner primers with BLAST (basic local alignment search tool) at NCBI (National center for biotechnology information) confirmed the 100% specificity of these primers only to *Leishmania* and therefore no amplification occurs with other organisms (Fig. 1b) [30]. But as shown in Fig. 1b, the region amplified by the inner primers are present in other kinetoplastids (eg: *Leptomonas* and *Crithidia*) with some changes in nucleotide sequences between those organisms.

### Sample size determination

The sample size required for the study was calculated according to following standard formulas, [TP + FN = Z^2^ × (SN(1-SN))/W^2^ and n = (TP + FN)/P where TP: true positive, FN: false negative, SN: lowest acceptable sensitivity of diagnostic test used; According to previously published data, STNPCR method was 100% sensitive for diagnosis ([28]. Therefore SN was taken as 98.0% for sample calculation, Z: normal distribution value at particular confidence interval (i.e. for 95%, Z = 1.96), P: leishmaniasis prevalence within the suspected group of patients (the clinically suspected group of CL patients referred to Department of Parasitology, Faculty of Medicine, University of Colombo); According to a recent study, the prevalence of suspected group of patients referred to the department was found as 86.4% [8]. Therefore *P* value was taken as about 80.0% for sample calculation, W: represents accuracy; A 5.0% of sensitivity of confidence interval is the standard value determined according to sample size calculation methods and n: sample size] [32]. TP + FN and n were calculated as 30.12 and 37.6 respectively. When *n* = 37.6 (approximately *n* = 38), total samples to be analyzed from a suspected population of leishmaniasis with 80.0% disease prevalence and with 98.0% expected sensitivity of the Mo-STNPCR method was about *n* = 70. A total of 70 (40 patients suspected for CL and 30 patients suspected for VL) patients were included in the study.

### Sample collection

Samples were collected from the patients referred to Department of Parasitology, Faculty of Medicine, University of Colombo after informed written consent. Laboratory confirmation of samples were done by LM, IVC [33] and/or CPCR [34]. The selected *n* = 30 CL and *n* = 10 VL patients confirmed for leishmaniasis were used for evaluating Mo-STNPCR method. The control group included NCL (*n* = 5 cutaneous TB and *n* = 5 leprosy), NVL (*n* = 10) and HC (*n* = 10). From the CL and NCL patients, lesion material (lesion aspirates or slit-skin scrapings, *n* = 25) and skin biopsies (*n* = 15) were collected. Bone marrow aspirates were collected from all VL and NVL patients. Peripheral blood (*n* = 5) or skin materials/skin cells collected by impression with a glass slide in non-invasive manner (*n* = 5) were collected from healthy individuals to normalize the effect of type of tissue.

### DNA preparation

DNA extractions from the samples were done using Qiagen mini DNA extraction kit in accordance with manufacturer’s guidelines. A minimum sample amount of 200 μl was aliquoted from body fluids such as blood, bone marrow, lesion aspirates or slit-skin scrapings. Twenty (20) μl of proteinase K and 200 μl of lysis buffer were added to each sample. Tubes were incubated in a water bath at 56 °C for 10 min following vortexing for 15 s. Body tissues (skin biopsies, about 2 mm sized) were subjected to additional tissue disruption (cut into small pieces) with the buffer provided by manufacturer. The lysis of body tissues/skin biopsies were cut into small pieces for facilitating complete lysis of the tissue with the buffer and proteinase K provided (Also if require, additional mechanical disruption using a homogenizer can reduce the lysis time of the sample. Complete lysis of cells is required to yield a high DNA yield since body tissues have an abundance of contractile proteins, connective tissue and collagen). After adding 20 μl of proteinase K, the samples and reagents were mixed by vortexing. Then they were incubated at 56 °C for 1–3 h until completely lysed. Occasional vortexing of the samples was done during the incubation period. An additional incubation was carried out for all body tissue samples at 70 °C for 10 min after mixing with 200 μl of lysis buffer.

All the lysed samples were mixed well by vortexing with 200 μl of absolute ethanol and transferred to DNA extraction columns. After centrifuging the columns at 8000 rpm for 1 min, two washing steps were carried out using 500 μl of each wash buffer provided by the manufacturer by centrifuging at 8000 rpm for 1 min and 12,000 rpm for 1 min respectively. Finally, the DNA samples were eluted from binding columns by adding 200 μl of elution buffer followed by 5 min incubation at room temperature and centrifugation at 8000 rpm for 1 min. The extracted DNA samples were stored at 4 °C in a refrigerator until further use. Long-term storage was done at -20 °C.

### Negative and positive controls for Mo-STNPCR

**Fig. 2:**
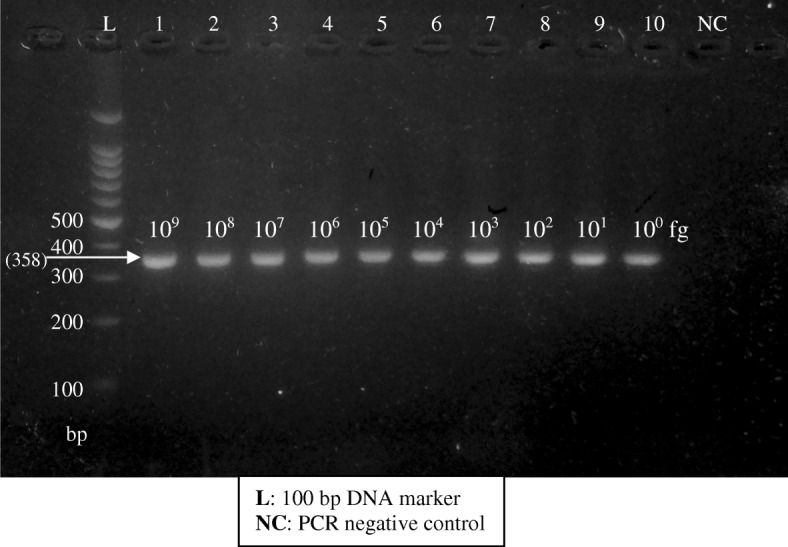
Gel image of sensitivity assay done using a dilution series of the positive control

Total genomic DNA extracted from a cell pellet of *L. donovani* (local reference strain of *Leishmania*) promastigote mass culture was used as the reference positive control. For the quality assurance of the new test, negative controls were run parallel to the positive control in each round of PCR. Few negative controls were used including PCR reactions containing only inner primers, only outer primers, both primers without template DNA and both primers with DNA coming from a negative extraction. These different negative controls further enhanced the quality of the test by excluding any non-specific amplifications, contaminations of reagents used for DNA extraction and PCR. The lowest detection limit of the Mo-STNPCR was tested using a standard series of a positive control with a known concentration. Ten-fold dilutions were prepared from the positive control which contained 1 μg to 1 fg of DNA and they were amplified using Mo-STNPCR (Fig. 2).

### Mo-STNPCR method

A mixture of inner primers including forward (P223: 5′-TCCCATCGCAACCTCGGTT-3′) and reverse (P333: 5′-AAGCGGGCGCGGTGCTG-3′) primers [18] with traces of bromophenol blue was prepared by adding 1:1:1 ratio of forward primer (10 μM), reverse primer (10 μM) and bromophenol blue [25]. A total of 3 μl of inner primers of the nested PCR were immobilized on to the inner side of cap of PCR tubes prior to adding PCR mixture to each tube. The immobilization was done by allowing the primers-bromophenol blue mixture to air dry at room temperature (24 °C) for about 2–3 h by keeping the cap of tubes open at a horizontal position on a PCR tube rack. These primer immobilized PCR tubes were stored at -20 °C until further use.

PCR reaction mixture was prepared by adding outer primers of the nested PCR (forward primer (10 μM), P221: 5′-GGTTCCTTTCCTGATTTACG-3′ and reverse primer (10 μM), P332: 5′-GGCCGGTAAAGGCCGAATAG-3′) [18, 34] at a ratio of 10:1 between inner primers: outer primers. PCR reaction was set up using the optimized volumes of PCR Taq master mix (abm/ Applied Biological Materials, Canada) and other reagents as shown in Table 1. The PCR programme was run for 15 cycles in first step and 35 cycles in second step (Table 2).

**Table 1:** Components of PCR master mix

PCR water	19.8 μl
2XPCR Taq master mix (abm, G013)	25.0 μl
Forward, outer primer (P221)	0.1 μl
Reverse, outer primer (P332)	0.1 μl
Template DNA	5.0 μl
Total	50.0 μl

**Table 2:**
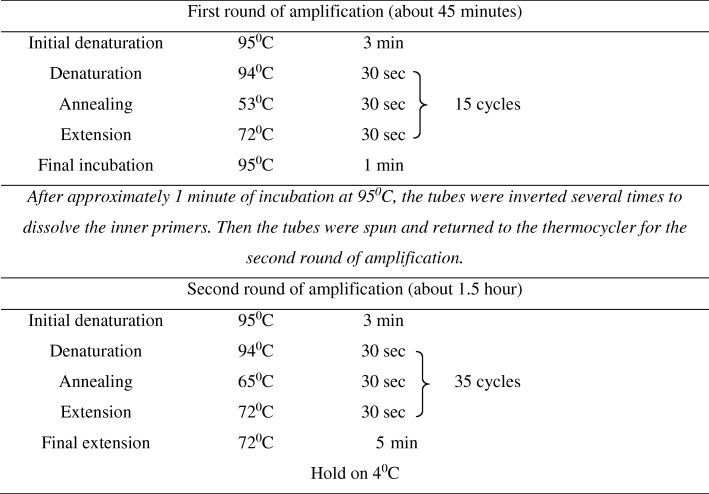
PCR programme for Mo-STNPCR

### Gel electrophoresis

Ten microliters of PCR products against the 100 bp DNA ladder were analyzed on 2.0% agarose gels consisted with ethidium bromide. DNA bands were visualized with an ultraviolet light transilluminator. Positive samples yielded a PCR product of 358 bp.

### Other techniques for comparison

As described above, lesion aspirates, slit-skin scrapings or skin biopsies collected from CL and NCL patients, bone marrow collected from VL and NVL patients and peripheral blood or skin materials collected from HC were analyzed and compared with LM, IVC and CPCR. All CL (*n* = 30), VL (*n* = 10), NCL (*n* = 10), NVL (*n* = 10) and HC (*n* = 10) samples were analyzed using LM. Also IVC was carried out for all CL, VL and NVL samples while all VL, NVL, HC and some CL (n = 10/30) samples were further confirmed with CPCR. In LM, Giemsa-stained smears were microscopically examined with 1000× magnification [33]. Minimum of 100 fields were observed before marked as negative. The micro-capillary culture method was used for IVC with complete M199 media (Gibco) [33]. IVC were observed until completion of two weeks from initial inoculation before marked as negative. CPCR was done using previously established standard protocols using P221 and P332 primers [34].

### Validation and data analysis

A sample was taken as *Leishmania* positive if it was positive by any one of the control tests performed viz. LM, IVC or CPCR. Reproducibility or repeatability of the assay was determined over one year period by performing a known positive and known negative samples in each round of Mo-STNPCR. Sensitivity, specificity, NPV and PPV were calculated using the standard 2 × 2 tables. Accuracy of the test was also determined using standard calculation methods (Accuracy = true positives and true negatives/total number of samples). To further validate the Mo-STNPCR, the assay results were compared with classical parasitological diagnostic methods used for diagnosis of leishmaniasis, LM and IVC [33] and CPCR [34]. It aided in the determination of usefulness of Mo-STNPCR as a diagnostic tool.

### Cost analysis for Mo-STNPCR method

Cost analysis per patient was carried out according to approved guidelines of basic cost accounting for clinical services [35]. Briefly, expenses for DNA extraction and Mo-STNPCR, laboratory consumables, chemicals and reagents were estimated according to their current cost in USD (Table 3). The expenses for laboratory personnel and equipment were not considered for the analysis.

**Table 3:** Details of items used for evaluating the per patient cost of Mo-STNPCR

Major steps of Mo-STNPCR	Considered items for per patient cost analysis of Mo-STNPCR
Sample collection	Sample collection tubes and other materials required for sample collection
DNA extraction	DNA extraction kits for sample, positive control and negative control
PCR procedure	PCR water, 2XPCR Taq master mix (abm, G013), outer primers (P221 and P332) and inner primer (P223 and P333), bromophenol blue
PCR product analysis	Gel running buffer, ethidium bromide, agarose
Other consumables and reagents	Micro-centrifuge tubes, PCR tubes, pipette tips, phosphate buffered saline, issuing laboratory reports

## Results

The lowest detection limit of DNA in the Mo-STNPCR method was 1 fg, which was the lowest tested concentrations (Fig. 2).

The analysis and comparison of other diagnostic methods (LM, IVC and CPCR) of leishmaniasis confirmed the diagnosis of leishmaniasis and verified the positive and negative results obtained for Mo-STNPCR (Table 4). The positivity rates of other diagnostic methods tested were 75% (*n* = 30/40) and 72.5% (*n* = 29/40) for LM (Table 5) and IVC respectively. Also the combined LM and IVC gave 87.5% (*n* = 35/40) positivity (Table 6). As compared to LM and IVC, Mo-STNPCR showed 100% (*n* = 40/40) positivity for the study group of leishmaniasis. Also all negative control samples including patients with other non CL skin diseases (NCL), patients with other non VL systemic diseases (NVL) and healthy individuals (HC) gave negative results in Mo-STNPCR.

**Table 4:** Diagnostic 2 × 2 table. Sensitivity, specificity, negative predictive value (NPV) and positive predictive value (PPV) of the Mo-STNPCR method were determined by comparing to conventional parasitological methods, LM, IVC and CPCR

		Combined laboratory results (LM, IVC and CPCR)			
		Positive	Negative	Total count	
Mo-STNPCR method	Positive	40	0	40	PPV = (40/40) × 100% = 100.0%
	Negative	0	30	30	NPV = (30/30) × 100% = 100.0%
	Total Count	40	30	70	
		Sensitivity = (40/40) × 100% = 100.0%	Specificity = (30/30) × 100% = 100.0%

**Table 5:** Diagnostic 2 × 2 table. Sensitivity, specificity, NPV and PPV of the Mo-STNPCR method were determined by comparing to LM

		LM			
		Positive	Negative	Total count	
Mo-STNPCR method	Positive	30	10	40	PPV = (30/40) × 100% = 75.0%
	Negative	0	30	30	NPV = (30/30) × 100% = 100.0%
	Total Count	30	40	70	
		Sensitivity = (30/30) × 100% = 100.0%	Specificity = (30/40) × 100% = 75.0%

**Table 6:** Diagnostic 2 × 2 table. Sensitivity, specificity, NPV and PPV of the Mo-STNPCR method were determined by comparing to combined LM and IVC results

		Combined laboratory results (LM and IVC)			
		Positive	Negative	Total count	
Mo-STNPCR method	Positive	35	5	40	PPV = (35/40) × 100% = 87.5%
	Negative	0	30	30	NPV = (30/30) × 100% = 100.0%
	Total Count	35	35	70	
		Sensitivity = (35/35) × 100% = 100.0%	Specificity = (30/35) × 100% = 85.7%		

The accuracy of Mo-STNPCR method was calculated as 100.0% where *n* = 40 true positives, *n* = 30 true negatives and *n* = 70 of total samples for CL, VL and control group together (Table 4). The Mo-STNPCR method was 100.0% repeatable. There was no difference between the results of Mo-STNPCR and combined LM, IVC and CPCR results of analyzed samples. But low positivity of other diagnostic tests as mentioned above confirmed the high sensitivity and accuracy of Mo-STNPCR method compared to those individual diagnostic methods. Per patient cost was calculated as 22 USD while the same was 3 and 6 USD for light microscopy and in-vitro culture respectively.

## Discussion

This study produced a fully sensitive diagnostic tool to detect leishmaniasis while enabling the exclusion of *Crithidia spp. and Leptomonas* spp. which are non-leishmanial pathogens that can complicate detection of *Leishmania spp*. in IVCs as well as in some widely used CPCR methods [34]. qPCR method is another PCR technique with high sensitivity and specificity. In qPCR, an intercalating dye or fluorescence probe are used for quantifying number of amplified DNA molecules using the strength of signal produced by them. Although qPCR is with high sensitivity and specificity and avoids the requirement for post amplification analysis of PCR, the disadvantages of qPCR which are the high cost, complexity and technically demanding nature limit its usage ([21]). qPCR is currently not available for diagnosis in all *Leishmania* diagnostic laboratories within the country. Mo-STNPCR can be carried out in conventional PCR machine and therefore it is highly applicable than qPCR for diagnosis of *Leishmania*.

CPCR amplifies a region of about 603 bp in ssurRNA of *Leishmania* genome [34, 36]. CPCR was approximately 95% (*n* = 38/40) sensitive for detection of leishmaniasis according to the past records (for a different sample group studied within the centre). Albeit it can amplify *Leishmania* species, *Leptomonas* and *Crithidia* species [30]. This is a major disadvantage of the CPCR since *Leptomonas* and *Crithidia* can co-exist with *Leishmania* and leads to misdiagnosis even if they are non-pathogenic to humans [37, 38]. In the absence of an indicative skin lesion and in the presence of non-specific clinical features in VL, skin tissue or bone marrow examination needs careful exclusion of these pathogens. This problem was overcome by designing a new primer pair, P223 and P333 which amplified a region between the amplicon of P221 and P332 as shown in Fig. 1 and thus they were used as inner primers of the nested PCR method described [30]. The region amplified by P223 and P333 is specific only to *Leishmania* genus and therefore it excludes the amplification of *Leptomonas* and *Crithidia* species according to the BLAST search carried out on published GenBank data. Therefore the NPCR done using P223 and P333 inner primers are 100% specific for detection of *Leishmania* parasites [18, 30].

STNPCR technique was also called as “drop-in/drop-out” nested PCR which consisted inner and outer primers with vastly different annealing temperatures thereby initially allowed only the outer primers to amplify and subsequently the inner primers to amplify the nested fragment [39–41]. Therefore this method restricted the selection of primers since it required significantly different annealing temperatures [42]. Also the different research groups tried to physically separate the first and second round amplifications by using different procedures with different materials such as a thin layer of mineral oil, agarose resin and trehalose matrix [43–45]. But these methods are cumbersome, required the use of specially designed reaction tubes and presence of extraneous materials might interfere with the PCR [42]. The inner primers showed activity (with the positive PCR bands) only where tubes were inverted and dissolve the inner primers fixed inside the lid. The monitoring of color change of reaction mixture [the mixture was converted to light blue color after dissolving with inner primers (mixed with bromophenol blue)] also re-confirmed the stability of inner primers inside the lid against possible evaporation and condensations.

STNPCR employed in this study has been successfully applied for detection of other pathogenic microorganisms such as *Schistosoma mansoni* [42, 46], *Plasmodium falciparum* [47], *Yersinia pestis* [48], dengue virus serotypes [26], *Vibrio cholera* O1 [27], *Leishmania chagasi* [25], Porcine Circovirus type 2 [28] and *Mycobacterium tuberculosis* [29], causing range of clinical conditions.

Current study used a new combination of previously established inner and outer primers and they were examined using modifications to the technique. The results of Mo-STNPCR showed high sensitivity, specificity (each 100.0%) and therefore a high accuracy for diagnosis of leishmaniasis. Furthermore, NPCR is likely to have limited use as a diagnostic tool due to carry over and cross-contaminations. Strict adherence to protocol from DNA extraction step onwards would ensure best results in Mo-STNPCR. Also there may be a loss of reactants due to same initial PCR mixture being used for both rounds of PCR in Mo-STNPCR method. However, the selection of a satisfactory primer ratio between inner and outer primers can ensure sufficient reactants until completion of the reaction. It is also important to avoid excessive primer concentrations to prevent creation of 603 bp band in second round of amplification and other non-specific PCR products [45].

## Conclusions

Late presentations, atypical presentations and chronic non-treatment responsive cases in an endemic setting limit the usefulness of clinical detection as well as microscopic or culture detection of leishmaniasis. Furthermore, VL detection requires tools that ideally diagnose 100% cases due to the essential needs of both death prevention and laboratory confirmation prior to introducing toxic and expensive treatment modalities. LM/IVC does not detect all cases. Even though such scenarios often constitute the minority in an endemic setting, establishing a diagnosis in all cases is necessary. In such a situation level of sensitivity become more important as compared to the cost of investigation, simplicity or the wider availability. The new Mo-STNPCR test described here can easily replace the CPCR with better results and may be used as a useful tertiary level assay for detection of all LM and IVC negative leishmaniasis cases in Sri Lanka and other leishmaniasis endemic settings.

## Data Availability

The data supporting the conclusions of this article are included within the article. Additional details are available from the corresponding author on reasonable request.

## Abbreviations

CL: Cutaneous leishmaniasis
CPCR: Conventional single-step PCR
DNA: Deoxyribonucleic acid
HC: Healthy individuals
ITS: Internal transcribed spacer
IVC: In-vitro culturing
kDNA: Kinetoplastid DNA
LM: Light microscopy
MCL: Muco-cutaneous leishmaniasis
Mo-STNPCR: Modified single tube-nested PCR
NCL: Non CL skin diseases
NPCR: Nested PCR
NPV: Negative predictive value
NVL: Non VL systemic diseases
PCR: Polymerase chain reaction
PKDL: Post-kala-azar dermal leishmaniasis
PPV: Positive predictive value
ssurRNA: Small subunit ribosomal RNA
VL: Visceral leishmaniasis
